# Analysis of multi drug resistant tuberculosis (MDR-TB) financial protection policy: MDR-TB health insurance schemes, in Chhattisgarh state, India

**DOI:** 10.1186/s13561-018-0187-5

**Published:** 2018-01-27

**Authors:** Debashish Kundu, Nandini Sharma, Sarabjit Chadha, Samia Laokri, George Awungafac, Lai Jiang, Miqdad Asaria

**Affiliations:** 1 International Union Against Tuberculosis and Lung Disease (The Union), South-East Asia Office, C-6, Qutub Institutional Area, New Delhi, 110016 India; 2 Department of Community Medicine, Maulana Azad Medical College, New Delhi, India; 3 Universite Libre de Bruxelles, Brussels, Belgium; 4 African Society of Laboratory Medicine; Ministry of Health, Cameroon, Yaoundé, Cameroon; 5 Center for Instructional Psychology and Technology, Faculty of Psychology and Education Science, KU Leuven, Leuven, Belgium; 6 Global Health and Development, Imperial College London; Centre for Health Economics, University of York, York, United Kingdom

**Keywords:** Multi-drug resistant tuberculosis, Health insurance, RSBY, Universal health coverage, Financial protection policy, Inequity, Kingdon’s multiple streams, Implementation, Poor, India

## Abstract

**Introduction:**

There are significant financial barriers to access treatment for multi drug resistant tuberculosis (MDR-TB) in India. To address these challenges, Chhattisgarh state in India has established a MDR-TB financial protection policy by creating MDR-TB benefit packages as part of the universal health insurance scheme that the state has rolled out in their effort towards attaining Universal Health Coverage for all its residents. In these schemes the state purchases health insurance against set packages of services from third party health insurance agencies on behalf of all its residents. Provider payment reform by strategic purchasing through output based payments (lump sum fee is reimbursed as per the MDR-TB benefit package rates) to the providers – both public and private health facilities empanelled under the insurance scheme was the key intervention.

**Aim:**

To understand the implementation gap between policy and practice of the benefit packages with respect to equity in utilization of package claims by the poor patients in public and private sector.

**Methods:**

Data from primary health insurance claims from January 2013 to December 2015, were analysed using an extension of ‘Kingdon’s multiple streams for policy implementation framework’ to explain the implementation gap between policy and practice of the MDR-TB benefit packages.

**Results:**

The total number of claims for MDR-TB benefit packages increased over the study period mainly from poor patients treated in public facilities, particularly for the pre-treatment evaluation and hospital stay packages. Variations and inequities in utilizing the packages were observed between poor and non-poor beneficiaries in public and private sector. Private providers participation in the new MDR-TB financial protection mechanism through the universal health insurance scheme was observed to be much lower than might be expected given their share of healthcare provision overall in India.

**Conclusion:**

Our findings suggest that there may be an implementation gap due to weak coupling between the problem and the policy streams, reflecting weak coordination between state nodal agency and the state TB department. There is a pressing need to build strong institutional capacity of the public and private sector for improving service delivery to MDR-TB patients through this new health insurance mechanism.

## Introduction

Public subsidies that target the poor and vulnerable are widely used in developing countries to increase their access to health care. Targeted subsidies can be provided through health insurance premiums, health equity funds, vouchers, conditional cash transfers; and are demand side health financing mechanisms for achieving universal health coverage (UHC) [1–3]. In many high and middle income countries, insurance based models are the main instruments used to ensure financial protection for the entire population, achieved through financial reforms in revenue collection, pooling and purchasing [4]. Such health financing reforms improve equity in the distribution of resources, leading to improvements in equity in utilization of services and financial protection [4] by ensuring robust implementation of the insurance schemes, acting as a vehicle for achieving universal health coverage (UHC).

In India, health system is pluralistic with asymmetric healthcare distributive network across public and private sector [5], with 66% of hospitals and 80% of ambulatory care provided by the private sector [6]. The high user fees charged by these private providers combined with low health insurance penetration have led to high levels of out-of-pocket (OOP) expenditure. These high OOP expenditures are particularly evident in the diagnosis and treatment of chronic diseases, often resulting in catastrophic health expenditure for poorer patients [5] thereby jeopardising India’s progress towards UHC. One such chronic disease disproportionately prevalent amongst the poor is Tuberculosis (TB) [7]. Looking at financial risk protection in relation to TB can therefore highlight important general lessons to inform decisions toward effective policy-making in the context of achieving UHC [8].

The World Health Organization recommends addressing poverty in national TB control programmes by promoting equity and pro-poor policies in disease prevention and control activities [9]. The high cost of treatment and the need to take medication over a long period of time, especially for multi-drug resistant TB (MDR-TB) patients, makes treatment less accessible for the poor [10]. Moreover, implementation gap previously shown in the literature appeared to lead to increased risk of incurring catastrophic expenditure due to TB [11]. In India, 84,000 multidrug-resistant (MDR)-TB cases are estimated to emerge annually among notified pulmonary TB cases [12], while a similar volume of cases are expected to be managed by the private sector but remain un-notified [13]. MDR-TB is forecasted to increase by 12% among incident TB cases in India, and additional control efforts are urgently required, beyond diagnosis and treatment of MDR-TB [14], to prevent increasing risk of incurring catastrophic expenditure due to MDR-TB. Disease burden due to TB in India is 3.27% (2.58% - 4.21%) of total DALY’s [15], which can be averted by expansion and robust implementation of tuberculosis services that are cost-effective in high-burden countries [16]. There is need to have greater emphasis on innovative patient support mechanism, which among many includes prevention of catastrophic health expenditure due to MDR-TB through health insurance mechanism [13].

Kingdon’s agenda-setting framework, suggests that when conditions in three streams: Problem, Politics and Policy, come together to bring an issue into the policy agenda, a window of opportunity arises for policy change [17]. The issue has to be seen to address a clearly defined problem (in the problem stream) and then a policy solution to the problem has to be available (in the policy stream) [18]. The political environment has to be favourable in addressing the problem (in the political stream). Such coupling creates an open policy window [17, 18]. In Chhattisgarh, a “tribal” state (as notified by the Government of India) in central India, similar coupling of Kingdon’s three streams for agenda setting had taken place that had led to the emergence of a financial risk protection policy for MDR-TB patients in the state. This is described as follows:

### Policy stream

In 2011, the Programmatic Management of Drug Resistant (PMDT) programme was launched in the State of Chhattisgarh as per the national PMDT policy expansion vision. The state attained full coverage for treating the MDR-TB ‘free of cost’ with centres established to identify Drug Resistant (DR) -TB in all the medical colleges of the state as of December 2012. As per the national PMDT policy, all MDR-TB patients have to undergo pre-treatment evaluations. The drug resistant TB patient should be hospitalized at the DR-TB Centre for a period of seven days to undergo pre-treatment evaluation for identifying those patients who are at a greater risk of adverse effects and to establish a baseline for monitoring, as the drugs for management of MDR-TB patients (2nd line anti-tuberculosis drugs) are toxic in nature [19]. Drug Resistant-TB centre is ideally established in the medical college hospital, a tertiary level health centre for pre-treatment evaluation, treatment initiation and management of side effects. During the same year, 2012, TB was made a notifiable disease by the Government of India [20].

### Problem stream

Drug-resistant TB is known to be fatal and is estimated to be 100 times more costlier to treat [21] than cases of drug-sensitive TB [22]. In the private sector, out of pocket (OOP) health expenditure by a MDR-TB patient due to user fees for staying in the hospital, laboratory investigations on account of pre-treatment and follow-up evaluations are estimated to be eighty times, three times and four times more expensive than in the public sector respectively. This will often force poorer households to incur catastrophic health expenditure leading to impoverishment if not protected by a financial protection mechanism [13]. User fees for laboratory investigations (both pre-treatment and follow-up investigations) can be catastrophic for a poor MDR-TB patient even in the public sector [13]. OOP health expenditure accruing to any household member with TB that exceeds one-fifth (20%) of household annual income is considered to be catastrophic for that household [23].

### Politics stream

**Fig. 1:**
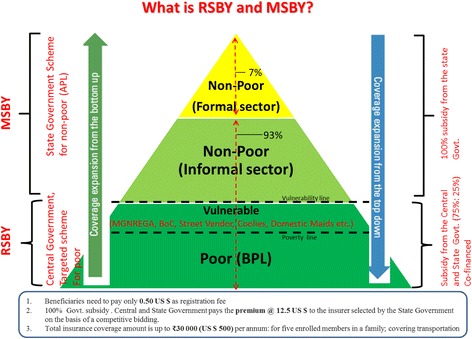
Universal Health Insurance Scheme (UHIS) as managed by RSBY and MSBY (Developed from *Cotlear* et al.*, 2015)*

The government of Chhattisgarh, envisages achieving UHC by improving the affordability, availability and accessibility of quality health care to every resident of the state [24]. To this end, the state had initiated universal health insurance scheme (UHIS) after announcement of this initiative from the Chief Minister, political head of the government in the state, in 2012. The UHIS provides health insurance coverage and protection to all people to fund their medical treatment on voluntary and hospitalization basis. It is managed by the National Health Insurance Programme, known as the “Rashtriya Swasthya Bima Yojana (RSBY)” Or social security scheme for a family with a maximum 5 household (HH) members who either live below the poverty line (BPL) or are members of specific categories of unorganized workers. These categories include - Mahatma Gandhi National Rural Employment Guarantee Act (MGNREGA) workers, Building and Other Construction (BOC) workers, Beedi workers, Domestic workers, Licensed Railway Porters, Street Vendors, Sanitation Workers, Mine Workers, Rickshaw Pullers, Rag Pickers, Auto and Taxi Drivers [25]. In addition to this Chhattisgarh state also provides the “Mukhyamantri Swasthya Bima Yojana (MSBY)” Or Chief Minister’s Health Insurance Scheme for every HH in the state, if not covered by RSBY. MSBY is also limited to a maximum 5 HH members, and consists mainly of HH who are above the poverty line (APL) [Fig. 1]. Chhattisgarh is the first state in the country to initiate the UHIS which provides health insurance coverage to all state residents to fund their medical treatment.

### Window of opportunity (coupling of problem and politics stream) for emergence of MDR-TB financial protection policy

Leveraging the opportunity for inter-sectoral collaboration, the State Tuberculosis (TB) Control Programme in Chhattisgarh facilitated partnership with RSBY and MSBY for Multi Drug Resistant TB (MDR-TB) patients in the year 2012 through creation of MDR-TB benefit packages for absorbing user fees for all pre-treatment evaluations, admissions, follow-up evaluations, ancillary drugs and nutritional support across all RSBY and MSBY empanelled network hospitals (both private and public) in the state [13]. The MDR-TB benefit packages are [Table 1]: 1) MDR-TB pre-treatment evaluation, 2) MDR-TB follow-up evaluation, and 3) MDR-TB hospital stay; an innovative financial protection mechanism to absorb OOP expenses incurred by MDR-TB patients from diagnosis to treatment completion across the public and private sector [13]. Overall objectives for establishing the MDR-TB benefits packages as a financial protection policy were to achieve equity in utilization of the packages in the sense that patients with equal needs should receive the benefit of the packages irrespective of their income (between poor and non-poor) [26]. Provider payment financial reform through fee for service was established by the creation of three MDR-TB benefit packages. Also, as per the insurance policy, the pre-treatment and follow-up packages are to be co-used with the hospital stay package, as utilization of the RSBY and MSBY health insurance scheme package for MDR-TB is based on hospitalization. The key financial reform steps taken for the MDR-TB patients in Chhattisgarh were:

1. Creation of RSBY and MSBY benefit packages [13] targeting the MDR-TB patients and integrating these packages with the list of other RSBY and MSBY disease packages in Chhattisgarh.
2. Piggy-backing on the already existing national health insurance programme - RSBY.
3. Contracting of the third party insurance agency (TPA) by the State Nodal Agency, to obtain pre-defined health services for the MDR-TB patients.

**Table 1:** Details of the innovative Rashtriya Swasthya Bima Yojna (RSBY) and Mukhyamantri Swasthya Bima Yojna (MSBY) MDR-TB benefit packages

MDR-TB benefit package name	Package details	Package Rate	Number of times/days claims can be processed (Package Cap)
Pre-treatment evaluations after diagnosis of MDR-TB	Chest X-ray, relevant haematological and biochemical tests: complete blood count (CBC), liver function tests (LFT), thyroid function tests (TFT), blood urea nitrogen (BUN), creatinine, urine (routine & microscopic), urinary pregnancy tests (UPT)	₹4000 (US$ 67^a^)	Once
Follow-up evaluation	Chest X-ray, relevant haematological and biochemical tests: CBC, LFT, BUN, creatinine, urine (routine & microscopic)	₹3300 (US$ 55)	Maximum five times for creatinine and all other tests for maximum of twice
Hospital stay	Bed charges, doctors’ consultation fees and any other additional/ancillary drugs	₹5600 (US$ 93 @ US$ 13/day)	Maximum 7 days’ stay on pro-rota basis

*MDR-TB* multidrug-resistant tuberculosis

Initial experience with such a collaboration between Revised National TB Control Programme (RNTCP) and the UHIS through creation of benefit packages for patients with MDR-TB shows that such partnership can be set up and can in principle act to reduce OOP expenditure [13]. Previous studies have highlighted the mechanism emphasizing collaboration between RNTCP and health insurance schemes (RSBY and MSBY) for MDR-TB patients [13]. Exclusionary process that operates at all steps of implementation of the RSBY scheme due to issues of awareness, enrolment, utilization, delay in reimbursements to providers and fraudulent practices have been widely studied [27–31]. However, none of the existing studies have reported on the variation and inequities in utilization for specific benefits packages within the insurance scheme. Therefore, the aim of this study is to whether or not the implementation of the MDR-TB health insurance packages is effective in –

- Benefitting those who needs them most, especially by equitable utilization of the packages by the poorest 20% quintile population [32];
- Improving the private sector involvement in RNTCP.

By addressing these questions new insights will be gained on implementation of RSBY and MSBY MDR-TB benefit packages for both poor and non-poor MDR-TB patients, paving efficient and feasible ways to support progress towards India’s journey to UHC.

## Methods

### Setting

The state of Chhattisgarh in central India (population 28 million, having 27 districts) has 80% of the population living in rural areas and 30% are considered “tribal”. Out of 29 states in India, Chhattisgarh is the 10th largest and 17th most populated state in the country. Backward class of population, namely Scheduled Tribes (ST) and Scheduled Castes (SC), constitute 31.8% and 12% of the state’s population respectively which belong to the most disadvantaged socio-economic groups in India [33]. The SC people are the one who were previously ‘untouchables’ and ST are community of people who lived in tribal areas (mainly forest) and are also known as ‘Adivasis’ [34]. Together the ST and SC population constitute 43% of the total population in the state [35] and have been traditionally marginalized. The state is also an insurgency hit (Left Wing Extremism or LWE) and poorest state in India, with 47.9% of people is living BPL, followed by 46.7% in Manipur and 45.9% in Odhisa. Of all states in India, the states of Chhattisgarh, Manipur, Odhisa, Madhya Pradesh, Jharkhand, Bihar and Assam figure among the poorest states where over 40% of people are below poverty line [36]. 24 out of 27 districts in Chhattisgarh are backward districts [37] with only three non-backward or economically rich districts. As per the National Sample Survey Organization (NSSO) nationwide household consumer expenditure survey, monthly per capita consumption expenditure (MPCE) is estimated to be below 10 US$ (₹ 600) per month in Chhattisgarh [38]. Besides, such socio-demographic characteristics, the state has higher number of health facilities in public sector than in private sector [39], and hardly any private health facilities in the tribal areas. Most of the RSBY and MSBY empanelled private health facilities are located in the urban areas [39].

#### How RSBY and MSBY health insurance schemes work?

Under the schemes each enrolled family is provided with a bio-metric smart card for paperless, cashless and portable transactions through smart cards. Each family is provided with a health insurance benefit of 500 US$ per family per annum on a family floater basis (upto 5 members in a family) and coverage of financial costs of the hospitalization expense. Hospitalization can be for both medical and surgical procedures (as per the predefined RSBY and MSBY package list for medical and surgical procedures). Conditions that are treated at home, congenital external diseases, drugs and alcohol induced illness, vaccination, war, nuclear invasion, suicide, naturopathy, Ayurveda, Unani and Siddha are excluded from the schemes (24). A key feature of RSBY health insurance scheme is portability - A beneficiary who has been enrolled in a particular district can use the smart card in any RSBY empanelled hospital across India. This makes the scheme truly unique and beneficial to the poor families who migrate from one place to the other [13, 25]. Beneficiaries of the scheme get cash less treatment in the government and private health institutions empanelled under the RSBY and MSBY as per their choice within the state and country. Additionally, transport expenses of ~ 2 US$ per hospitalization is paid to the beneficiary subject to a maximum of ~ 17 US$ per year per family. The beneficiaries need to pay only 0.5 US$ as registration fee for a year while the Central and State Government pays the fixed premium (12.5 US$) as per their sharing ratio (between Centre and State, 75:25 for RSBY, 0:100 for MSBY) to the private insurer selected by the state government on the basis of a competitive bidding [Fig. 1].

**Fig. 2:**
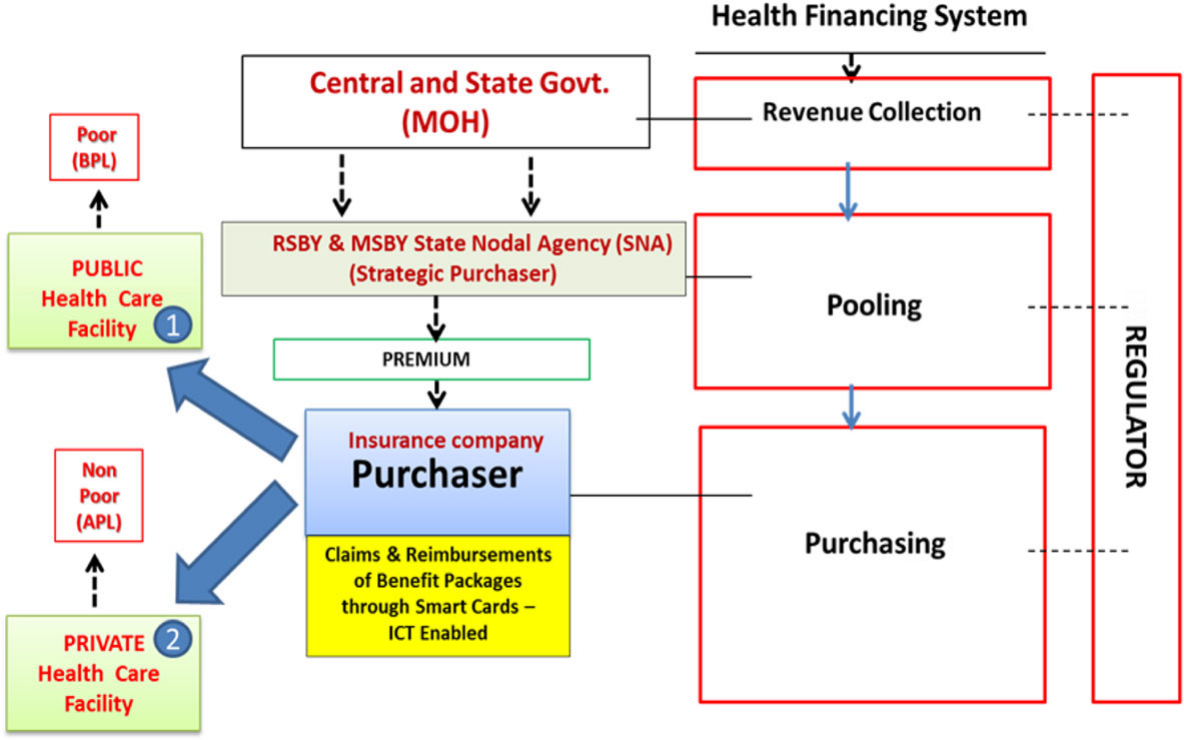
RSBY and MSBY, a business model, an organization as a nexus of contracts/ institutions (Developed from Kutzin J. Health financing for universal coverage and health system performance: concepts and implications for policy, Bulletin of World Health Organization, 2013;91(8): 602–11; and concept of organization as a nexus of contracts by Bruno Messen, Institute of Tropical Medicine, Antwerp. 2016)

In India, 93% of workforce is in informal sector [18] where there is no formal employee and employer relationship arrangements, having both poor and non-poor [Fig. 1]. RSBY scheme for social security for the poor receives complete subsidy from the central and state Government. However, in case of MSBY, 100% subsidy is provided by the State Government of Chhattisgarh for non-poor [Fig. 1]. In every state, the State Government sets up a State Nodal Agency (SNA) that is responsible for implementing, monitoring supervision and part-financing of the scheme by coordinating with a private insurance company, hospital, district authorities and other local stake holders. Therefore the scheme has been designed as a business model, an organization with nexus of contracts or institutions, for the social sector with incentives built-in for each stakeholder [Fig. 2]. Design of this business model can be linked to the function of the health financing systems [4], wherein Central and State Governments collect revenue from the general tax system, which is pooled at the state level by the RSBY and MSBY state nodal agency to pay premiums to the selected private insurance company. The private insurance company reimburses the claims of the benefit packages from both private (accessed mainly by non-poor: APL) and public health facilities (accessed mainly by poor: BPL and unorganized workers) [Fig. 2]. So there is transfer of pooled resources to public and private service providers for giving fee for service [40] to the beneficiaries by the purchasers - state nodal agency and the insurance company, with weak regulation by the Government. Strategic purchasing through output based payments (a lump sum fee is reimbursed as per the MDR-TB package rates) to the providers (public or private health facilities empanelled under the schemes) by the third party health insurance agency (TPA) is the provider payment method used in RSBY and MSBY Universal Health Insurance Scheme. This business model design is seen as conducive both in terms of expansion of the scheme as well as for its long run sustainability [Fig. 2].

### Data collection

Primary claims data on the uptake of ‘RSBY and MSBY MDR-TB’ packages under the routine national health insurance programme setting were collected from the server, accessible at the RSBY and MSBY State Nodal Agency of Directorate of Health Services, Raipur, from January 2013 to December 2015. This information was shared electronically with the principal investigator by the State Nodal Agency.

### Data variables

The pre-defined data variables on patient code, package name, hospital name, hospital type (Public or Private), registration and discharge descriptions, scheme code (RSBY or MSBY), patient characteristics – age, sex, APL, BPL, unorganized worker, district and claim status, were collected.

### Data processing and analysis

In total, 1159 records were checked by going through to identify any errors. 40 records for the Nuapada district were excluded as this district in not part of Chhattisgarh state. Non-MDR-TB cases were also excluded from the data analysis. A final clean dataset was imported to Epi-Info version 7.1.5.2 software (CDC Atlanta, USA) for analysis. We performed descriptive statistics analysis using means, median and proportions. Where comparisons were needed, we used the Chi square test with α set at 5%. Trends in the utilization of the packages from 2013 to 2015 in public and private sectors were derived to bring out and compare differences in utilization. An extension of Kingdon’s Multiple Streams for policy implementation framework [41] was applied to understand the implementation gap in the financial risk protection policy for MDR-TB patients.

## Results

### Utilisation of claims in public vs private sector

**Fig. 3:**
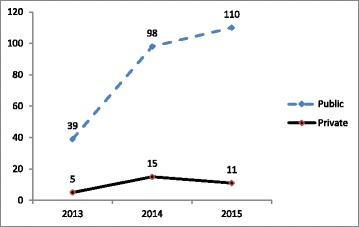
Pooled (RSBY and MSBY) MDR-TB hospital stay package utilization in the public and private sector from 2013 to 2015

**Fig. 4:**
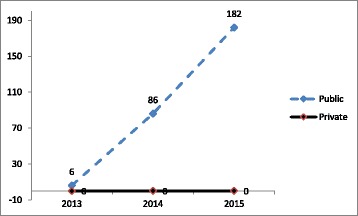
Pooled (RSBY and MSBY) MDR-TB follow-up package utilization in the public and private sector from 2013 to 2015

A total of 1159 RSBY and MSBY MDR-TB package claims were utilized by the beneficiaries [median age 43 years (IQR 30.5–55.5)], 67% claims from males, between 2013 to 2015. A total of 1044 (90.1%) claims were utilized by the beneficiaries in public health facilities as compared to 115 (9.9%) claims utilization in the private sector [Table 2]. 627 (54%), 278 (24%) and 254 (22%) claims were respectively processed under MDR-TB pre-treatment evaluation package, MDR-TB hospital stay package and MDR-TB follow-up evaluation package [Table 3]. Trends in utilization of MDR-TB hospital stay and follow-up evaluation packages showed better utilization of claims from the public sector [Figs. 3 and 4]. No claims were utilized under the MDR-TB follow up evaluation package from the private sector [Fig. 4].

**Table 2:** Key characteristics of the beneficiaries of all MDR-TB Packages (2013–2015)

Key Characteristics	RSBY	MSBY	Total
	*N* = 911 (%)	*N* = 248 (%)	*n* = 1159 (%)
1. Age			
< 15	46 (5.1)	14 (5.7)	60 (5.2)
15–34	239 (26.2)	80 (32.3)	319 (27.5)
35–54	373 (49.9)	91 (36.7)	464 (40.0)
55^+^	253 (27.8)	63 (25.4)	316 (27.3)
2. Sex			
Male	624 (68.5)	154 (62.1)	778 (67.1)
Female	287 (31.5)	94 (37.9)	381 (32.9)
3. Socio-economic status			
Poor (1 + 2):	826 (90.7)	64 (25.8)	890 (76.8)
Non Poor (APL)	85 (9.3)	184 (74.2)	269 (23.2)
4. No. of claims utilized in public and private health facilities by the beneficiaries			
Public	822 (90.2)	222 (89.5)	1044 (90.1)
Private	89 (9.8)	26 (10.5)	115 (9.9)

**Table 3:** Claim utilization status by poor and non-poor beneficiaries in public and private sector as per the MDR-TB package types (2013–15)

Type of MDR-TB Package	Claim Utilization		
	Poor – N (%)	Non-Poor – N (%)	Total, n = 1159 (%)
a) MDR-TB Pre-treatment evaluation package utilization in			
Public	433 (87.6)	110 (82.7)	543
Private	61 (12.4)	23 (17.3)	84
Total	494 (100)	133 (100)	627 (54)
b) MDR-TB hospital stay package utilization in:			
Public	185 (88.9)	62 (88.6)	247
Private	23 (11.1)	8 (11.4)	31
Total	208 (100)	70 (100)	278 (24)
c) MDR-TB follow-up evaluation utilization in:			
Public	188 (100)	66 (100)	254
Private	0	0	0
Total	188 (100)	66 (100)	254 (22)

### Utilisation of claims by poor vs non-poor beneficiaries

**Fig. 5:**
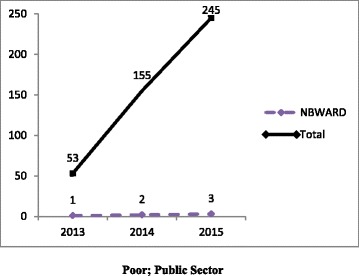
Disaggregated pooled (RSBY and MSBY) data on claims utilization of MDR-TB pre-treatment evaluation package by poor in the public sector from 2013 to 2015

**Fig. 6:**
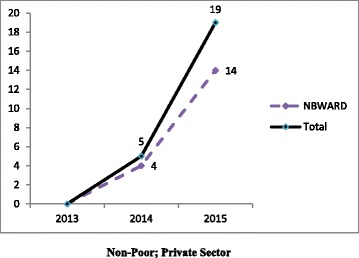
Disaggregated pooled (RSBY and MSBY) data on claims utilization of MDR-TB pre-treatment evaluation package by non-poor in the private sector from 2013 to 2015

Pooled (RSBY and MSBY) data on claims utilization of MDR-TB pre-treatment evaluation package showed that in the public sector, poor beneficiaries utilized five times more claims in 2015 than in 2013 mostly from the backward districts [Fig. 5]. In the private sector, non-poor beneficiaries utilized nineteen times more claims in the year 2015 compared to 2013 [Fig. 6]. Claims were utilized by non-poor fourteen times more in 2015 than in 2013 from non-backward or economically rich districts [Fig. 6]. In 2015, claims (313) surpassed the MDR-TB cases notified (215).

### Factors associated with the use of RSBY-MDR TB pre-treatment evaluation package

In bivariate analysis, the odds of one-time use of RSBY pre-treatment evaluation package in non-poor patients was found to be 0.04 [95% CI (0.02–0.07, *p* < 0.0001)] times less in comparison with the poor. This association remained statistically significant in multivariate analysis [Odds ratio: 0.03, 95%CI (0.01–0.05)]. Age, sex, district type and the type of institution had no significant association with the use of RSBY-MDR TB package. The results of bivariate and multivariate analysis are presented in Table 4.

**Table 4:** Factors associated with the use of RSBY MDR-TB Pre-Treatment Evaluation Package by MDR-TB beneficiaries

Variable	Bivariate analysis		Multivariate analysis	
	Odds Ratio, OR (95% CI)	*p*-value	Odds Ratio, OR (95% CI)	p-value
Age in years				
0–14	Ref			
15–34	0.88(0.36–2.19)	0.786	0.62 (0.20–1.98)	0.423
35–54	1.55 (0.62–3.83)	0.344	1.06 (0.33–3.40)	0.921
≥ 55	1.18 (0.47–2.98)	0.719	0.61 (0.18–2.01)	0.418
Sex				
Male	0.95 (0.63–1.43)	0.815	0.97 (0.55–1.71)	0.910
Female	Ref			
Socio-Economic Status (SES)				
Poor	0.04 (0.02–0.07)	< 0.0001	0.03 (0.01–0.05)	< 0.0001
Non-Poor	Ref			
District Sub-types				
Most Backward & Left Wing Extremist Districts(MBLWE)	0.61 (0.26–1.41)	0.247	1.38 (0.63–3.01)	0.426
Backward & Left Wing Extremist (BLWE)	0.94 (0.48–1.85)	0.868	Ref	
Backward Districts (BWARD)	0.85 (0.41–1.77)	0.66	1.36 (0.69–2.67)	0.372
Non Backward Districts (NBWARD)	Ref		0.74 (0.18–3.03)	
Hospital Types				
Private	1.15 (0.63–2.10)	0.635	1.77 (0.59–6.12)	0.364
Public	Ref			
Year				
2015	3.09 (1.79–5.34)	< 0.0001	5.64 (2.57–12.39)	< 0.00001
2014	2.07 (1.14–3.75)	0.016	1.76 (0.79–3.91)	0.169
2013	Ref			

## Discussion

RSBY and MSBY MDR-TB benefit packages were designed for financial risk protection of MDR-TB patients and to have equity in utilization of packages. These are the main goals of the health financing systems [40]. In the next section, extension of Kingdon’s Multiple Streams (Problem, Policy and Politics streams) framework [41] is used to discuss the utilization of RSBY and MSBY MDR-TB benefit packages in terms of equity in benefitting the poor from these packages.

### Problem stream

#### MDR-TB pre-treatment evaluation package vs. follow-up evaluation package

We found wide variations in claims utilization under the three MDR-TB packages with highest utilization in MDR-TB pre-treatment evaluation package - 627 (54%) and lowest utilization in MDR-TB follow-up evaluation package - 254 (22%). This finding indicates compromise in continuity of care, wherein follow-up evaluation is of paramount importance to monitor the progress of the treatment until the patient is cured. Low utilisation of follow-up evaluation package could also be attributed to its low package cost, and hence, is less attractive for processing claims under this package in both public and private sector.

#### Public vs. private

A substantial difference in terms of the total number of claims processed between the public and private sector was noted with much greater involvement of public health facilities in the financial risk protection mechanism for MDR-TB patients. Public health facilities seemed to outperform the private health facilities, which can be attributed to a bigger number public health facilities presence in rural and tribal areas, and hence their empanelment by the state health insurance schemes. This could be one of the possible reasons for low utilisation of claims by the beneficiaries in the private sector, unlike in majority of the states in India where almost half of the TB patients are accessing treatment in the private sector [42]. Chhattisgarh is an exceptional state where presence of public health infrastructure is higher than the national average [43], as majority of tribal districts in the state are insurgency hit [44] without much presence of private health facilities. The private sector is present mainly in urban cities and remains reluctant to move to remote or rural areas as it can make more profit by being in urban areas [39]. Establishing linkages between MDR-TB packages and the universal health insurance scheme (RSBY and MSBY) was an attempt to invent newer ways of public private partnership that would engage and leverage the involvement of private sector healthcare providers in MDR-TB care on a national scale in India. It was envisioned that implementing this health insurance model for MDR-TB patients could go a long way towards averting the majority of OOP expenditure in the private sector, especially by linking diagnosis and supply of drugs for MDR-TB with the national TB control programme of Government of India [13]. Effective public health programme linkage with the public and private sector is of paramount importance not only for financial risk protection of MDR-TB patients, but also for comprehensive control of TB in the community.

#### Poor vs non-poor

We found inequities in utilizing the packages under the RSBY and MSBY schemes by the non-poor and poor, which corroborate with evidence that voluntary health insurance schemes create similar inequities [45]. The non-poor MDR-TB patients were better able to access the private sector than the poor for utilizing claims under the MDR-TB pre-treatment evaluation package with an increasing trend and with drainage of public subsidy to the empanelled private health facilities. We also found gender inequity in utilising the claims as 67% of claims were utilised by the males. These disparities in utilizing the claims, which are unequal and inequitable, indicate lapse in proper implementation of MDR-TB benefit packages.

### Policy stream

#### Policy vs practice

Variations in utilization of the MDR-TB benefit packages by public and private sector were also observed. Firstly, not a single claim was processed under the MDR-TB follow-up evaluation package from the private sector. A MDR-TB patient requires minimum of eleven follow-up evaluations during the course of MDR-TB treatment as per the national Programmatic Management of Drug Resistant Tuberculosis (PMDT) guidelines [19]. Low utilization of MDR-TB follow-up evaluation package suggests that either the follow-up evaluations are not being done as per the schedule under the health insurance mechanism or not properly carried out under the routine programme setting or any other cause, and this needs to be investigated further. As per the policy of the benefit packages, its utilization under RSBY and MSBY schemes requires a hospital stay [24]. RSBY and MSBY MDR-TB packages are applicable for MDR-TB patients who are diagnosed as ‘MDR-TB’ cases by a RNTCP certified or any recognized laboratory on hospitalization basis. Ambulatory care is yet to be included and implemented in the mainstream health insurance [13]. However, utilization of MDR-TB hospital stay package was found to be sub-optimal 278 (24%). These aforementioned variations in utilizing the packages reflect on the weak implementation of MDR-TB benefit packages. The implementation gap previously shown in the literature lead to increased risk of incurring catastrophic expenditure due to TB [11]. Earlier studies on the mechanism of health insurance linkage with the TB control programme had recommended awareness campaigns, training and capacity building of joint programme staff for the success of this linkage [13]. Studies have suggested that key strategy to improve utilization of the RSBY scheme is by ensuring that the adequate information on entitlements and benefits reaches marginalized beneficiaries through proper awareness raising measures [13, 27–30].

### Politics stream

The political stream is present, but is loosely coupled with the problem and policy streams. At the time of agenda setting (Kingdon 1992), state level politics (Chief Minister’s political will to promote UHIS) influenced the formulation of the RSBY and MSBY MDR-TB financial protection policy. Similar influence was lacking in the implementation phase, as the implementation part of the programme was typically left to the programme officers.

#### Strengths and weaknesses of this study

There are important points that merit discussion on the strengths and weaknesses of this study. This is the first study which looked in detail how RSBY and MSBY MDR-TB benefit packages were used in the state of Chhattisgarh, India, disaggregated by poor and non-poor; in public and private sector; and across economically backward and rich districts, post its implementation suggesting weakness in it. We used fully electronic means of primary data collection and analysis. However, this study had certain limitations. Firstly, since the analysis is based on review of quantitative data received from State Nodal Agency (SNA), we do not know the quality of service received and patient satisfaction in utilising RSBY and MSBY MDR-TB packages. Secondly, we didn’t have data on MDR-TB cases from private sector and MDR-TB treatment outcomes from public and private sector. Hence, we don’t know impact of the intervention on adherence of MDR-TB treatment. To address these shortcomings future mixed methods (using both quantitative and qualitative) research for evaluating this intervention and for assessing quality of services for MDR-TB patients can be proposed [46]. Finally we only had data for MDR-TB patients and not all patients enrolled in the RSBY and MSBY schemes. So we were unable to undertake multivariate analysis to fully explore the differences in uptake of the packages between the different groups and facilities controlling for the various confounding factors. Examining this full dataset using multivariate regression techniques would be a key area for further research.

### Conclusion and recommendation

An implementation gap was observed, reflecting weak coordination between state nodal agencies and the state TB department in the Chhatisgarh MDR-TB programme. This creates an opportunity for a policy entrepreneur to emerge, seize the window of opportunity and advocate change. Variations and inequities in utilization of MDR-TB packages; and low utilization of follow-up evaluation package could be the consequences of weak implementation of the MDR-TB benefit packages in the state of Chhattisgarh. Public health efforts should be consolidated in strengthening the vast presence of public health facilities in the state through proper institutional arrangements by establishing linkages with the national TB control programme for improving service delivery to the MDR-TB patients in order to achieve universal health coverage. Proper implementation of MDR-TB benefit packages through the health insurance mechanism could go a long way in contributing towards achieving universal health coverage in India, Sustainable Development Goal (SDG) 3 of the United Nations that articulates to ensure healthy lives and promote well-being for all, and progress towards achieving the end-TB strategy target of zero catastrophic costs due to TB by 2035. Complete engagement of the national programmes from the stage of planning to execution, and periodic programme review is necessary to ensure feasible and successful implementation of a policy intervention [47].

The following steps are recommended before scaling up this innovative initiative for financial protection of MDR-TB patients across the country based on literature review - 1) Creating awareness [13, 27–30] to empower the MDR-TB patients on their entitlements primarily at the health facilities level which are empanelled in the health insurance schemes. This can be achieved by adequate counselling from the field staff to the patients for reducing variations and inequities in utilisation of packages. 2) Joint programme review meetings [13, 27, 48] for ensuring close monitoring of MDR-TB and health insurance programme (UHIS), identifying and addressing critical bottlenecks, and to remove inequities by strengthening the public sector and regulating the private sector, are to be convened by the local stewards [27, 49] at state and district levels. 3) Training and capacity building of both RNTCP and RSBY and MSBY State Nodal Agency staff in the state [13] by the master trainers of these programmes for correct identification, enrolment, utilization and passing the benefits of the packages to the beneficiaries.
